# The dynamic process and microscopic mechanism of extraordinary terahertz transmission through perforated superconducting films

**DOI:** 10.1038/srep15588

**Published:** 2015-10-26

**Authors:** J. B. Wu, X. Zhang, B. B. Jin, H. T. Liu, Y. H. Chen, Z. Y. Li, C. H. Zhang, L. Kang, W. W. Xu, J. Chen, H. B. Wang, M. Tonouchi, P. H. Wu

**Affiliations:** 1 Research Institute of Superconductor Electronics (RISE), School of Electronic Science and Engineering, Nanjing University, Nanjing 210093, China; 2 School of Electronic and Electrical Engineering, University of Leeds, Leeds LS2 9JT, United Kingdom; 3 Key Lab of Optical Information Science and Technology (MOE), Institute of Modern Optics, Nankai University, Tianjin 300071, China; 4 Laboratory of Optical Physics, Institute of Physics, Chinese Academy of Sciences, Beijing 100190, China; 5 National Institute for Materials Science, Tsukuba 305-0047, Japan; 6 Institute of Laser Engineering, Osaka University, 2-6 Yamadaoka, Suita, Osaka 565-0871, Japan

## Abstract

Superconductor is a compelling plasmonic medium at terahertz frequencies owing to its intrinsic low Ohmic loss and good tuning property. However, the microscopic physics of the interaction between terahertz wave and superconducting plasmonic structures is still unknown. In this paper, we conducted experiments of the enhanced terahertz transmission through a series of superconducting NbN subwavelength hole arrays, and employed microscopic hybrid wave model in theoretical analysis of the role of hybrid waves in the enhanced transmission. The theoretical calculation provided a good match of experimental data. In particular, we obtained the following results. When the width of the holes is far below wavelength, the enhanced transmission is mainly caused by localized resonance around individual holes. On the contrary, when the holes are large, hybrid waves scattered by the array of holes dominate the extraordinary transmission. The surface plasmon polaritions are proved to be launched on the surface of superconducting film and the excitation efficiency increases when the temperature approaches critical temperature and the working frequency goes near energy gap frequency. This work will enrich our knowledge on the microscopic physics of extraordinary optical transmission at terahertz frequencies and contribute to developing terahertz plasmonic devices.

The greatly enhanced transmission of light through subwavelength hole array in metal film, *i.e*., extraordinary optical transmission (EOT) phenomenon, was first observed by Ebbesen *et al*.^1^ This discovery greatly contributes to the study of how light interacts with subwavelength metallic structures and exhibits many promising applications in photonics such as biosensors, solar cells and nanolithography^2^,^3^,^4^,^5^,^6^. Some researchers attribute this phenomenon to the presence of surface plasmon polaritons (SPPs), a type of surface wave propagating along the metal-dielectric interface^1^,^7^,^8^,^9^. However, it is questionable that whether SPPs play an essential role in this physical process. More recently, by employing microscopic model, it is demonstrated quantitatively that two distinct surface waves scattered by subwavelength holes, SPP and quasi-cylindrical wave (QCW), both contribute to the enhanced transmission^10^,^11^,^12^,^13^.

Superconductors have been proposed as a potential plasmonic medium for EOT at terahertz (THz) frequencies below their energy gap frequencies because of the high kinetic energy of superconducting carriers. Their inherent low Ohmic loss and good tuning property imply the potential applications in a variety of plasmonic devices and transmission lines^14^,^15^,^16^,^17^,^18^,^19^. In addition, nonlinear surface Josephson plasmon wave is predicted to exist in anisotropic layered superconductor and to exhibit fascinating electromagnetic effects^20^,^21^,^22^,^23^,^24^. Superconducting films or Josephson junction stacks patterned with a regular array of micron-scale holes has attracted much attention because the presence of holes has strong influence on the critical current, critical magnetic field and vortex motion in superconducting film^25^,^26^,^27^,^28^,^29^. In recent years, it is demonstrated that the enhanced THz transmission through arrays of subwavelength holes in superconducting films exhibit a unique tuning property^14^,^15^,^16^,^17^,^18^. However, whether SPPs are excited on the surface of superconducting films and the microscopic mechanism of EOT phenomenon at THz frequencies remains unknown.

This paper reports a comprehensively study of the excitation and propagation of surface waves on perforated superconducting films by both experiments and theoretical analysis of microscopic model. In the experiments, the dynamic process that the THz pulse transmits through superconducting subwavelength hole array is observed by time domain spectroscopy. In the theoretical analysis, we adapt the microscopic hybrid wave (HW) model, a comprehensive model that has been used to clarify the microscopic physics of EOT at visible wavelength^10^,^11^,^12^,^13^,^30^. The temperature dependent transmission spectra are produced by computation using HW model, which highly match the experimental data. The result clearly demonstrate the contribution of HW including both SPPs and QCW to the EOT phenomenon. Furthermore, the paper also presents a theoretical interpretation of the tuning of resonance frequency.

## Results

**Figure 1:**
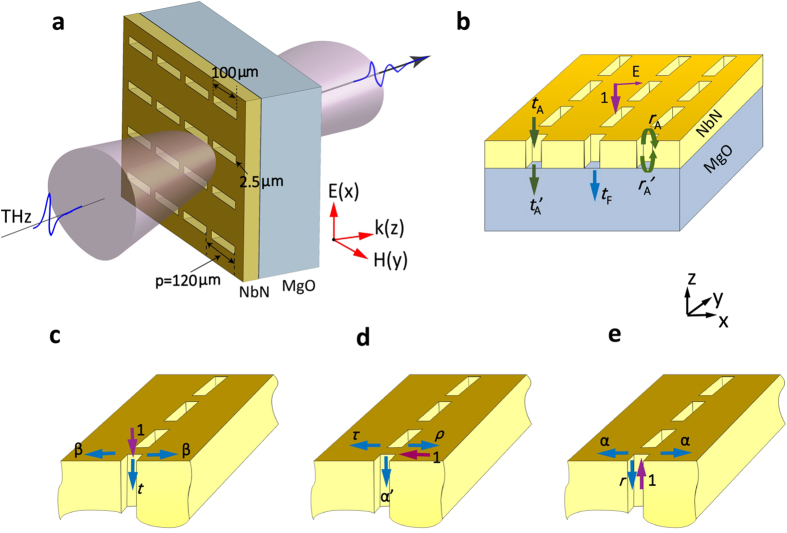
The diagram of superconducting subwavelength hole array and elementary HW scattering processes involved in EOT phenomenon. (**a**) The diagram of the transmission of pulsed THz radiation through W2.5. The hole length and width of W2.5 are 100 and 2.5 μm respectively. The periodicity of the hole array is 120 μm. (**b**) The transmission and reflection of the fundamental Bloch mode at the interface of NbN/air and NbN/MgO. (**c**) An incident transverse magnetic polarized (magnetic vector along *y* axis) plane wave being scattered by the one-dimensional hole array. (**d**) The scattering by hole array under illlumination of the SPP mode. (**e**) The fundamental Bloch mode of the hole array. (see Methods for the definition of scattering parameters of *α*, *α*′, *β*, *t*, *r*, *ρ*, and *τ* and transmission or reflection coefficients of *t*_*A*_, *t*_*A*_*'*, *t*_*F*_, *r*_*A*_, and *r′*_*A*_).

To investigate the EOT through the perforated superconducting films, a series of arrays of superconducting NbN rectangular holes on MgO substrate were designed and fabricated. The samples all possess the same periodicity of 120 μm. The holes in the samples are of equal length of 100 μm, and different width of 2.5, 4, 8, 15, and 30 μm (denoted by W2.5, W4, W8, W15 and W30, respectively in the sequel). As shown in Fig. 1(a), the THz pulses transmitted through the samples at different temperatures were measured utilizing THz time domain spectroscopy (TDS) system. In our experiments, the 1 mm-thick MgO substrates were chosen, so the time window of transmitted pulse without Fabry–Perot (F-P) interference was expanded to 20 ps. The obtained transmission spectra were correspondingly more accurate compared with former studies in which 0.5 mm-thick MgO substrates were used^16^. The microscopic HW model was used in the theoretical study of the microscopic physics of EOT phenomenon at THz frequencies (see more details in Methods). Figure 1(b–e) illustrate the elementary HW scattering processes involved in this phenomenon.

### Dynamic process of EOT

**Figure 2:**
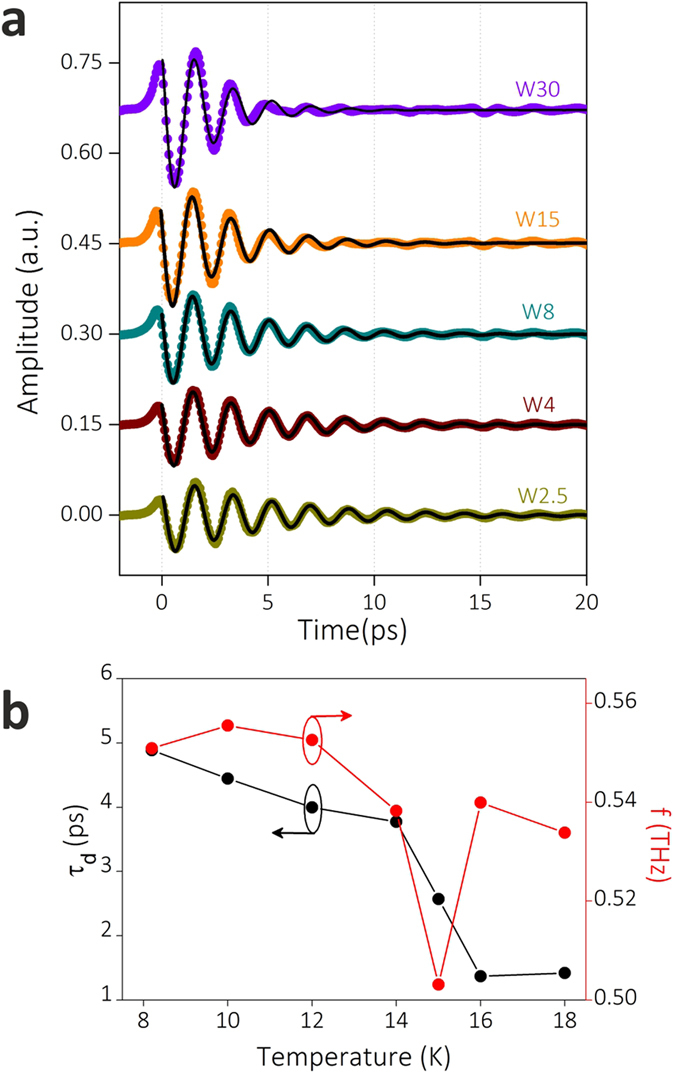
The time domain profiles of transmitted pulses through superconducting subwavelength hole arrays. (**a**) The measured time domain profiles (dots) of THz pulses through W30, W15, W8, W4, and W2.5 at 8.2 K and the corresponding fitted curves (black lines). (**b**) The decay time *τ*_*d*_ (black dots) and oscillation frequencies *f* (red dots) as a function of temperature which are obtained from the fitted curves of time domain profiles of W2.5.

The dynamic process of transmission of THz pulse through the perforated superconducting films at 8.2 K can be obtained from the picosecond-resolved time domain profiles. As shown in Fig. 2(a), the periodic and damped oscillations are observed for all of five superconducting samples. These time domain profiles are fitted by the damped sinusoidal waveform formula:


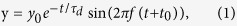


where *τ*_*d*_ is the decay time, *y*_0_ is the initial amplitude, *f* is the oscillation frequency and *t*_0_ is the initial time delay. As illustrated in Fig. 2(a), the fitted curves are in good accordance with the measured data, indicating that the enhanced transmission through the perforated superconducting films is a resonant transmission process. When the THz pulse is incident on the perforated superconducting film, the energy of electromagnetic waves at the frequencies satisfying the resonance condition is stored in the structured film. Then, the stored energy is gradually dissipated owing to Ohmic loss of superconducting film, or is converted to the outgoing THz wave through the interaction with the subwavelength holes.

For W2.5, the resonant transmission process is the most persistent and the oscillation lasts for more than 20 ps (Fig. 2(a)). As for the transmitted pulse through perforated gold film having the same geometry (shown in Supplementary Fig. 1(a)), the oscillations after 10 ps are less remarkable and the *τ*_*d*_ extracted from time domain profiles is much smaller. This difference is attributed to the remarkably lower Ohmic loss of superconducting NbN film than that of gold film. As NbN film goes into superconducting state, the transmitted THz pulses through W2.5 experience a remarkable change. The *τ*_*d*_ and f of W2.5 as a function of temperature are extracted and plotted in Fig. 2(b). In superconducting state, since the Ohmic loss in the resonant transmission process is greatly reduced*, τ*_*d*_ is remarkably higher than that in normal state. The *f*, i.e., the resonant transmission frequency, is observed to be tuned by temperature. As sample enters superconducting state, *f* sharply goes down to the lowest value, and then gradually increases with decreasing temperature. In addition, we notice that the fitted parameter of *t*_0_ changes from −0.217 ps at 18 K to −0.709 ps at 8.2 K. The difference in *t*_0_ corresponds to about 0.55*π* phase delay, which is mainly owing to the greatly enhanced kinetic inductance of NbN film in superconducting state^31^.

### Microscopic model analysis

**Figure 3:**
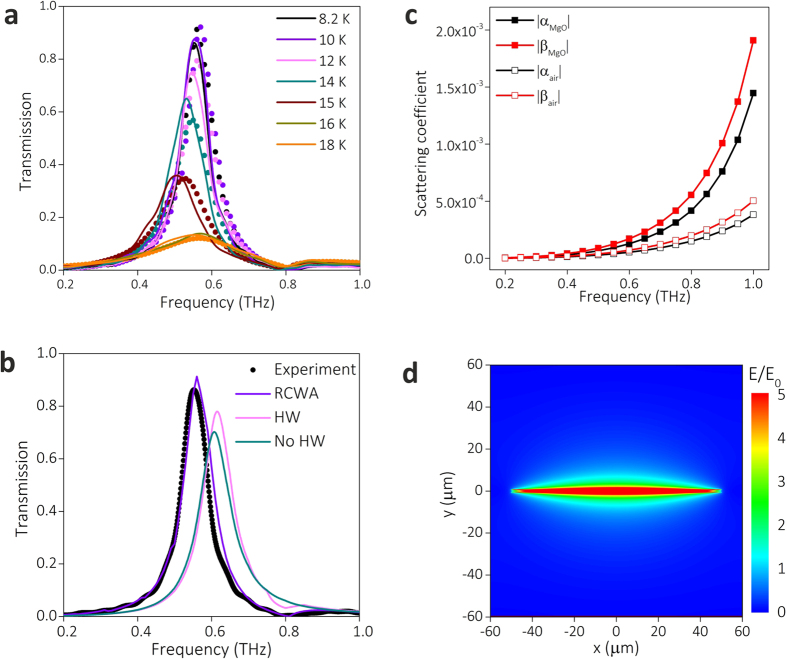
Comparison of the experimental data, HW model predictions, and RCWA computation data for the THz transmission spectra of W2.5. (**a**) The transmission spectra of W2.5 at different temperatures obtained from measurement (solid lines) and calculation using RCWA (dots). (**b**) The transmission spectra of W2.5 at 8.2 K obtained from the measurement, and calculation using RCWA, HW model and No-HW model. (**c**) The calculated absolute value of scattering coefficients of *α* and *β* at the NbN/air and NbN/MgO interfaces as a function of frequency at 8.2 K. (**d**) The simulated normalized electric field (*E/E*_0_) distribution at the interface of NbN/MgO at 8.2 K, where *E* is the calculated electric field excited around the hole and *E*_0_ denotes the average electric field of incident plane wave.

The measured THz transmission frequency domain spectra of W2.5 at different temperatures are plotted in Fig. 3(a). The tuning behaviours of peak amplitude and resonance frequency are consistent with our previous work^16^. The hole area of W2.5 only takes 1.7% of the total area, and the transmission peak reaches as high as 86.2% at 8.2 K. Correspondingly, the normalized transmission coefficient, which is the ratio of energy transmitted through the sample to the energy incident on the holes at the transmission peak frequency, is 50.7, is much larger than that of perforated gold film (see Supplementary Note 1).

To study the microscopic mechanism of EOT through the structured superconducting films, the microscopic HW model is used to calculate the EOT spectra. The microscopic model as well as the experimental data are validated using the rigorous coupled wave analysis^32^,^33^ (RCWA) (see more details in Methods). The results of RCWA are consistent with the measured transmission spectra as illustrated in Fig. 3(a). In the following, we compare the measured transmission spectra at 8.2 K and the corresponding computation results using HW model and RCWA. In Fig. 3(b), the results using HW model (pink) reproduce the transmission spectra obtained from calculation (purple) and measurement (black).

It is worth noting that there is a blue-shift of the peak position predicted by the HW model. This is mainly owing to the simplification used in the HW model, in which only the least-attenuated fundamental waveguide mode of the hole is taken into account while other high-order modes are all neglected^34^,^35^. Since the thickness of NbN film (200 nm) is much smaller than the free space THz wavelength, neglecting the transmittance of high-order modes can bring some error in the calculation using HW model.

To study the role of HWs in EOT phenomenon, we develop a No-HW model that neglects the contribution of the HW (including SPP and QCW) in the above microscopic HW model. Using No-HW model, we also calculate the transmission spectrum and plot it (green) in Fig. 3(b) for comparison. The calculated spectrum using No-HW model is quite close to that using HW model, indicating that both SPP and QCW do not play a dominant role in EOT through W2.5. To explain this phenomenon, we analyse the calculated scattering coefficients for the elementary scattering processes in the HW model. As Fig. 3(c) illustrates, the excitation coefficients of HW (*α* and *β*) are quite low. As the hole width of W2.5 (2.5 μm) is much smaller than the free space THz wavelength, the HW is hard to be excited through scattering of incident wave. Therefore, the contribution of HW to the enhanced transmission, which is represented by the second term in Eqs (5) and (6), is much smaller compared with the contribution of direct transmittance (*t*) and reflectance (*r*).

Based on above analysis, we can draw a conclusion that the enhanced transmission for W2.5 does not arise from collective resonant excitation of HWs, but it is mainly attributed to the localized resonance around individual hole. This result is consistent with theoretical study on optical transmission through single rectangular hole in perfect conductor film, in which the greatly enhanced transmission peak is predicted to appear around the cut-off wavelength of waveguide mode of the hole^36^. Since the normalized transmission coefficient of W2.5 is remarkably high and *τ*_*d*_ is large, it can be inferred that the quality factor of localized resonance around each hole is very high for W2.5 in superconducting state. To prove this, we simulate the electric field distribution using finite-difference time-domain (FDTD) method. As shown in the Fig. 3(d), the electric field around the rectangle hole (*E*) is greatly enhanced compared with the average electric field of incident wave (*E*_0_), indicating that strong resonance is excited around the rectangle hole.

**Figure 4:**
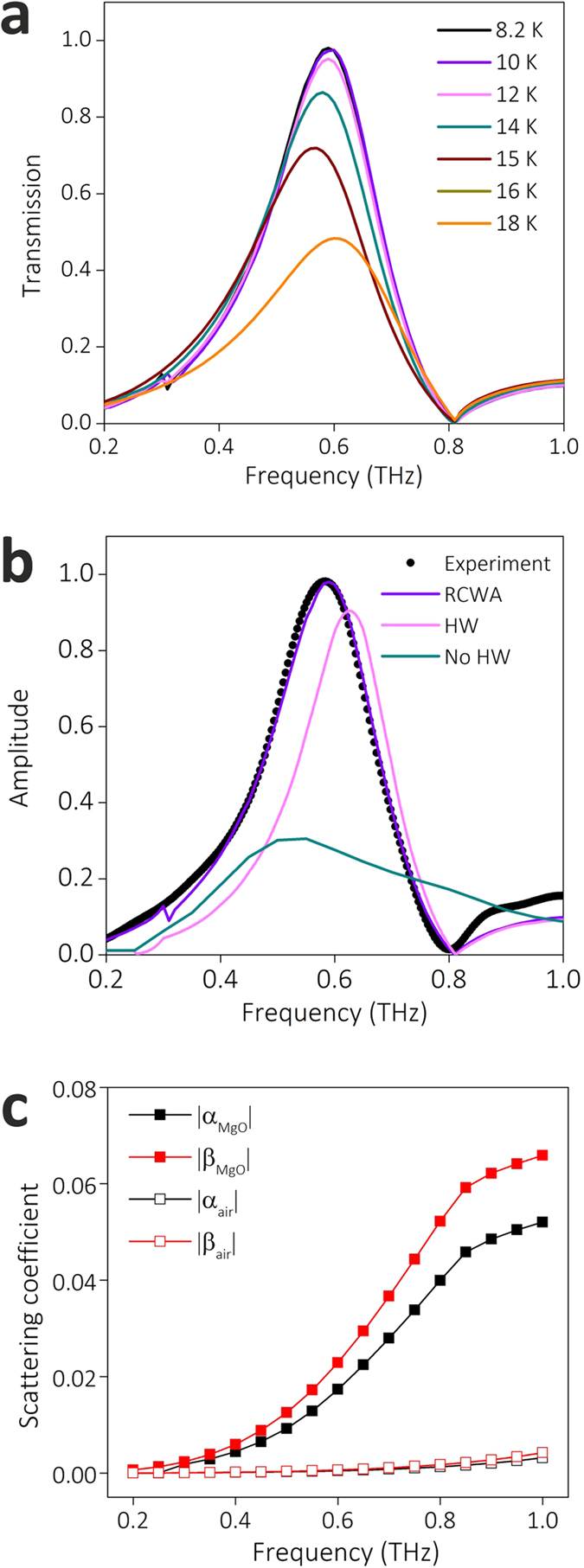
Comparison of the experimental data, HW model predictions, and RCWA computation data for the THz transmission spectra of W30. (**a**) The calculated transmission spectra of W30 at different temperature using RCWA model. (**b**) The comparison of transmission spectra at 8.2 K obtained from measurement, calculation using RCWA, HW model, and No-HW model. (**c**) The calculated absolute value of scattering coefficients of *α* and *β* at the interface of NbN/air and NbN/MgO as a function of frequency at 8.2 K.

If the length and width of holes are both comparable with wavelength of impinged wave, impinged wave will be strongly scattered by the holes and HWs will be launched. To explore the involvement of HWs in EOT, we perform microscopic HW model analysis for W30. As plotted in Fig. 4(a), the calculated transmission spectra using RCWA model are in accordance with measured transmission spectra of our previous work^16^. The contribution of HWs is assessed by comparing the measured spectra and calculation results using microscopic model. As shown in Fig. 4(b), the experimental results (black) and rigorous results using RCWA (purple) are well reproduced by the calculated spectrum using HW model (pink), but they are obviously underestimated in the calculated spectrum using No-HW model (green). The peak values of calculated transmission spectra using HW and No-HW model are 0.90 and 0.31, respectively. The large difference suggests that HWs play an important role in the EOT for W30. As shown in Fig. 4(c), it is observed that |*α*| and |*β*| at the MgO/NbN interface of W30 are two orders higher than those of W2.5, while these coefficients at air/NbN interface are still low, indicating that sufficient HWs are excited at MgO/NbN interface and involved in the EOT.

### Excitation of SPP on the surface of superconducting film

**Figure 5:**
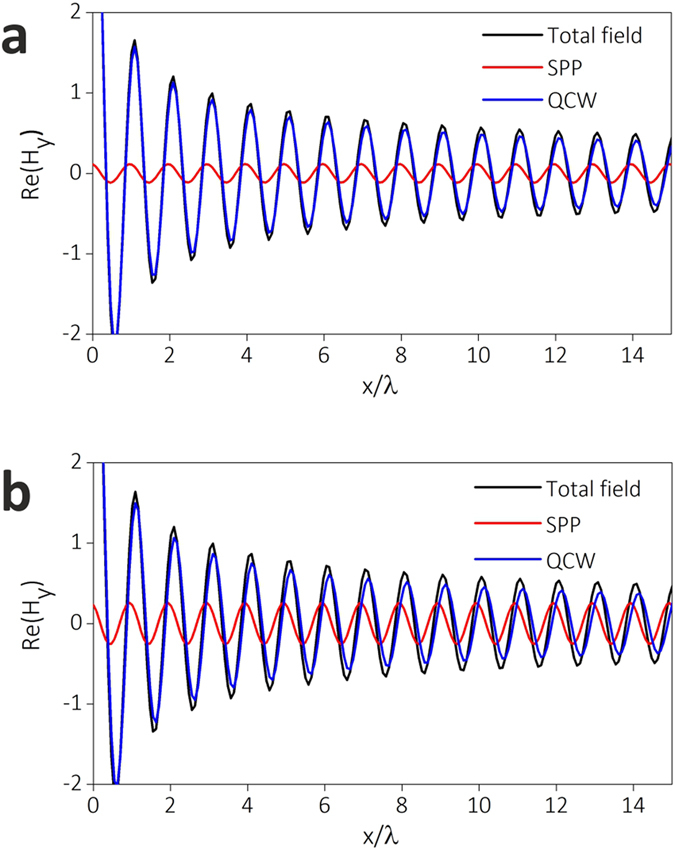
Surface waves generated at the interface of NbN and MgO at different working frequencies (*f*) and temperatures (*T*). (**a**) *f* = 0.6 THz, *T* = 8.2 K. (**b**) *f* = 1 THz, *T* = 14 K. The magnetic field is normalized at the interface. Total field (black), SPP (red), and QCW (blue) represent the field of HW, SPP, and QCW respectively.

In THz spectral region, metal can be viewed as perfect conductor, so SPP cannot be launched on the surface of metallic film. In order to clarify whether SPP is excited at the surface of superconducting NbN film, we calculate the excitation coefficients of HW, SPP and QCW at the interface of NbN/MgO at different frequencies and temperature. At 8.2 K and 0.6 THz, the SPP is launched at NbN/MgO interface as illustrated in Fig. 5(a), proving that superconductor is a plasmonic medium at THz frequencies. In spite of that, the ratio of SPP to QCW is quite low, which is mainly because the absolute value of the permittivity of NbN at THz frequencies is much larger than that of MgO^11^,^37^. For 0.6 THz radiation, the complex permittivity of NbN at 8.2 K is −99479 + *i*1493. Correspondingly, the calculated localization length of SPP in *z* direction (vertical to interface) toward the side of MgO is 2.56 mm, about 5 times of the free space wavelength, which means that the field of SPP cannot be strongly confined at NbN/MgO interface. As the temperature of sample approaches *T*_*c*_ and the working frequency approaches energy gap frequency, the absolute value of complex permittivity decreases, so the SPP excitation efficiency increases. In Fig. 5(b), the proportion of SPP field in the total scattered field at 14 K and 1.0 THz is over twice larger than the proportion in Fig. 5(a).

### Frequency tuning property

The tuning of resonance frequency is a unique property for superconducting plasmonic structures. The phenomenological Fano model, in which the resonance frequency is determined by the coupling of SPP resonance and localized resonance mode around each hole, explains the shift of resonance frequency^16^,^38^,^39^. However, the microscopic origin of the frequency tuning property is still unknown.

**Figure 6:**
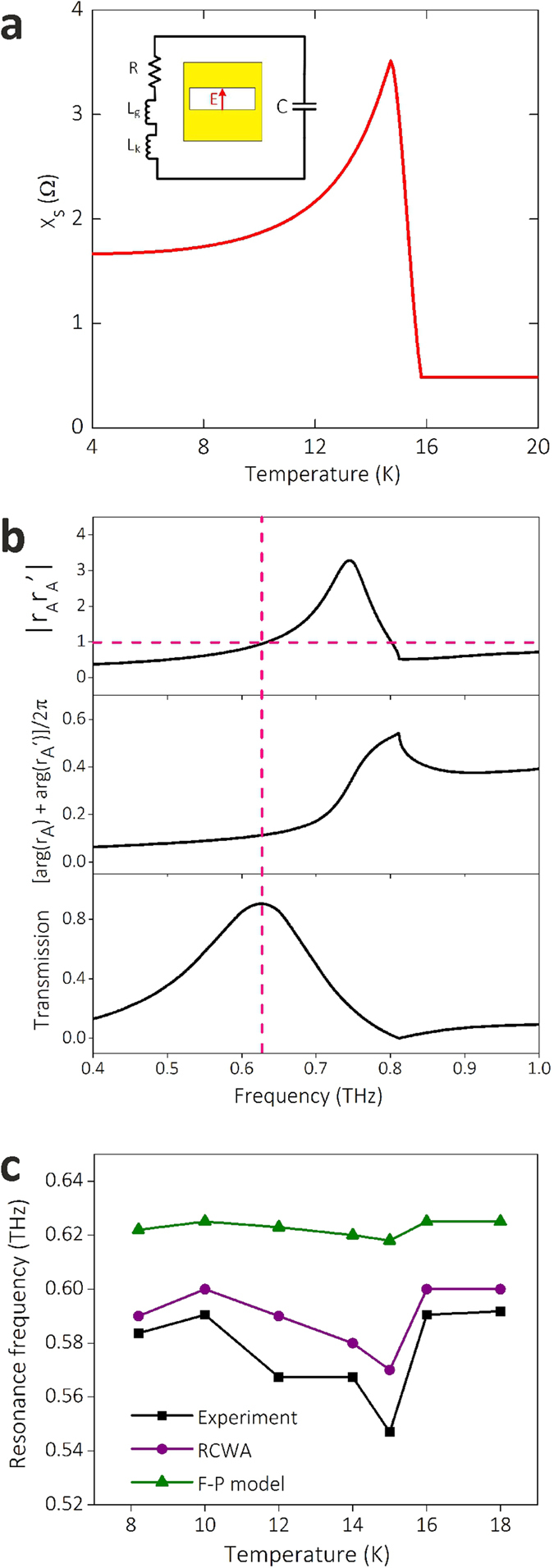
The tuning of resonance frequencies of EOT through superconducting subwavelength hole array. (**a**) The temperature dependence of surface reactance of NbN film at 0.6 THz. The equivalent circuit model of localized resonance around an individual hole is plotted in inset. **(b**) The condition of resonant transmission for W30. The top and centre graphs show the modulus and phase of *r*_*A*_*r*_*A*_*'* at 8.2 K obtained using HW model. The graph in the bottom shows the calculated transmission spectra using HW model. (**c**) The temperature dependence of the resonant transmission frequency for W30 obtained from measurement, and calculation using RCWA model and F-P model.

For sample having small holes such as W2.5, the resonance frequency is mainly determined by the waveguide mode of individual hole, as HWs are almost not involved in EOT process. In that case, the frequency tuning property of W2.5, similar as that of superconducting metamaterials, can be interpreted using an equivalent circuit model, in which the temperature dependent kinetic inductance of superconducting film is taken into account^40^,^41^. In the equivalent RLC circuit describing the localized resonance around a rectangular hole (inset of Fig. 6(a)), *C* represents the geometric capacitance, *L*_*g*_ and *L*_*k*_ are the geometric and kinetic inductance, and *R* is the Ohmic resistance. Correspondingly, the resonance frequency of waveguide mode is *f* ≈ (1/2π)[(*L*_*g*_* + L*_*k*_)*C*]^−1/2^. The non-monotonic change of *L*_*k*_ with temperature shown in Fig. 6(a), results in the non-monotonic change of resonance frequency.

For W30, conversely, HWs play a dominant role in the resonant transmission process, so the interaction of holes cannot be ignored. In that case, we study whether the resonant transmission peak is due to the constructive interference of HWs launched by each *y*-periodic chain of holes (see the coordinate in Fig. 1(b)), as this resonance condition is applicable for the EOT at optical frequencies^10^,^12^,^13^. Through a quantitative analysis, we find that the resonant transmission through W30 is not determined by the constructive interference of HWs, but still satisfies the F-P resonance condition expressed in Eq. (4)^35^. In Eq. (4), the value of *t*_*F*_ is maximized when the denominator in the right-hand side of Eq. (4) is minimized. At THz frequencies, the NbN hole depth (*h* = 200 nm) is much smaller than the free space wavelength, which results in exp(i2*k*_0_*n*_*FM*_*h*) ≈ 1, where *k*_0_ = 2*π*/*λ* is the free space wave number and *n*_*FM*_ is the complex effective refractive index of the fundamental mode. Therefore, the transmission peak position is determined by *r*_*A*_*r′*_*A*_. To satisfy the resonance condition, |*r*_*A*_*r′*_*A*_| ≈ 1 and arg(*r*_*A*_*r*_*A*_*'*) = arg(*r*_*A*_) + arg(*r*_*A*_*'*) ≈ 2 *mπ*, where arg represents argument, and *m* is an integer. We calculate the *r*_*A*_*r*_*A*_*'* as a function of frequency at 8.2 K and plot it together with the transmission spectra. As illustrated in Fig. 6(b), the resonant peak of EOT appears when |*r*_*A*_*r′*_*A*_| ≈ 1 and arg(*r*_*A*_) + arg(*r′*_*A*_) is close to 0. With the increase of temperature, *r*_*A*_*r′*_*A*_ experiences an obvious change, resulting in the movement of resonance frequency. Figure 6(c) shows the resonance frequency of W30 as a function of temperature obtained from measurement (black), calculation using RCWA (purple), and calculation using F-P model (green). The calculated temperature dependence of resonance frequency using F-P model has the same trend as experimental data and computation results using RCWA, despite that there is some difference in tuning range. The difference is mainly due to the simplification in F-P model in which high-order evanescent modes of hole array are omitted^42^,^43^.

## Discussion

The mechanism of the enhanced transmission through superconducting hole array is different from the well accepted mechanism of resonant excitation of surface waves at optical frequencies. However, the enhanced transmission phenomena can still be called EOT. The general definition of EOT is that the transmittance of hole array is larger than the transmittance of an individual hole^44^. As shown in Figs 3(b) and 4(b), the calculated transmittance of NbN hole array using HW model which takes into account the contribution of HWs to EOT is larger compared with the transmittance of individual hole in the array (No-HW model) which omits the interaction of the holes with the scattered HWs. In addition, the difference of the ratio of the film thickness to free space wavelength, can be used to explain their differences in resonant transmission condition between EOT through structured superconducting film at THz frequencies and EOT through structured metallic film at optical frequencies. Based on HW model, the *t*_*F*_ in Eq. (4) should reach the maximum at the transmission peak position. For the structured metallic film at optical frequencies, the hole depth (i.e. thickness of the metal film) is usually comparable with the free space wavelength, and *n*_*FM*_ is nearly a pure imaginary number^34^,^42^. Correspondingly, |exp(i*k*_0_*n*_*FM*_*h*)| in Eq. (4) is much smaller than 1. To reach the maximum, the absolute values of *t*_*A*_, *t*_*A*_*'*, *r*_*A*_ and *r*_*A*_*'* should be as large as possible. Based on the Eqs (5) and (6), the transmission peak needs to satisfy the phase-matching condition^12^:


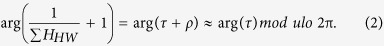


In other words, EOT phenomenon at optical frequencies is caused by the collective resonant excitation of HWs. For the structured superconducting film at THz frequencies, conversely, *h* is much smaller than the free space wavelength, so exp(*i*2*k*_0_*n*_*FM*_*h*) ≈ 1. Therefore, the transmission peak position is not determined by Eq. (2), but F-P resonance condition expressed in Eq. (4) is still applicable if HWs are involved in EOT process.

Based on theoretical analysis and experimental results, the SPPs can be launched on the surface of perforated superconducting film. That is a hallmark of plasmonic medium. In contrast, on the surface of structured metallic film at THz frequencies, only the spoof SPPs, *i.e*., QCWs in microscopic HW model, can be launched^45^,^46^,^47^. Though the behaviours of QCWs resemble the SPPs, the radiation loss of QCWs is much higher. As shown in Fig. 5, the amplitude of QCWs attenuates with an asymptotic behaviour |*x*|^−1/2^ (*x* is the propagation distance) because of their inherent radiation loss^11^. On the contrary, SPPs can propagate along the surface of superconductor without radiation loss. Therefore, the propagation length of SPPs on the surface of superconducting film is much larger than that of QCWs. The calculated propagation length of SPPs at 0.6 THz and 8.2 K on the smooth surface of NbN is 17.2 m, implying the prospect of superconductors in fabricating THz plasmonic transmission lines and circuits.

## Conclusion

In summary, the EOT through superconducting NbN subwavelength hole array at THz frequencies is comprehensively studied both experimentally and theoretically. The measured time resolved transmitted THz pulse illustrates the resonant transmission process. The microscopic mechanism of EOT through superconducting subwavelength hole array and the frequency tuning property are clarified using microscopic HW model. At THz frequencies, EOT phenomenon has been demonstrated on perforated films of other materials, like semiconductors^48^ and metals^49^. The microscopic HW model used in our calculation is also applicable for other plasmonic medium, therefore, it offers an effective way to expand our understanding on EOT phenomenon at THz frequencies. Furthermore, this study will contribute to developing novel THz plasmonic devices and waveguides.

## Methods

### Device fabrication and measurement

The samples were made from 200 nm-thick NbN films, which were deposited onto 1 mm-thick MgO substrates (single polished, <100> orientation) using RF magnetron sputtering. The measured critical temperature (*T*_*c*_) of NbN film was 16 K. The subwavelength hole array was patterned on the film using conventional photolithography. Then, the NbN film without protection of photoresist was etched down to MgO substrate using the reactive ion generated by a mixture of CHF_3_ and SF_6_ (20:20 sccm).

The samples were mounted in a continuous flow liquid helium cryostat, which was installed in the THz TDS system, for cryogenic THz transmission spectra measurement. The THz pulses of energy about 0.12 pJ/pulse and peak electric field amplitude far below 1 kV/cm are generated by photoconductive switch. The transmitted THz pulses through the samples were measured in a temperature range of 8.2–300 K. The diagram of W2.5 and the transmission of THz pulse through W2.5 are plotted in Fig. 1(a).

### Microscopic HW model

The elementary HW scattering processes involved in EOT phenomenon are shown in Fig. 1(b–e). The zeroth-order power transmittance of the perforated superconducting film can be expressed as


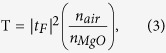


where *t*_*F*_ is the transmission coefficient of the zeroth order plane wave as illustrated in Fig. 1(b), *n*_*air*_ and *n*_*MgO*_ are the refractive index of air and MgO substrate. Under the assumption that the transmission of light is mediated by the least-attenuated fundamental mode of subwavelength hole array, *t*_*F*_ can be expressed with a F-P equation^10^,^42^,


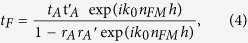


where *n*_*FM*_ is the complex effective refractive index of the fundamental mode, and *h* is the thickness of NbN film. As shown in Fig. 1(b), *t*_*A*_ is the transmission coefficient from incident plane wave to the fundamental mode of NbN hole array at the air/NbN interface, *t*_*A*_*'* is the transmission coefficient from the fundamental mode to the zeroth-order plane wave at the MgO/NbN interface, *r*_*A*_ and *r*_*A*_*'* are the reflection coefficients of fundamental mode of hole array at the NbN/air and NbN/MgO interface, respectively. Within the HW model, *t*_*A*_ and *r*_*A*_ at the NbN/air interface can be expressed as,


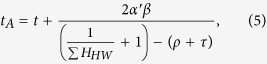



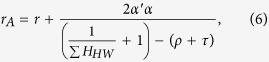


where *∑H*_*HW*_ represents a lattice summation of HW field at multiples of the period^12^. This HW field is composed of the field of a SPP and a QCW,
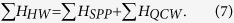


The scattering parameters in the elementary processes shown in Fig. 1(c–e) are defined as follows: *α*, the coupling coefficient from the fundamental hole-chain mode to the HW; *α*′, the reciprocal coupling coefficient from the HW to the fundamental hole-chain mode; *β*, the launching coefficient from incident plane wave to the HW; *t*, the transmission coefficient from the incident plane wave to the fundamental hole-chain mode; *r*, the reflection coefficient of the fundamental hole-chain mode; *ρ*, the reflection coefficient of the HW at a chain of holes; *τ*, the transmission coefficient of the HW at a chain of holes.

The transmission and reflection coefficients *t*_*A*_*'* and *r*_*A*_*'* at the NbN/MgO interface can be expressed similarly as *t*_*A*_ and *r*_*A*_ in the HW model. Here, it is worth mentioning that the transmission coefficient from an incident plane wave to the fundamental mode of NbN hole array and its reciprocal transmission coefficient from the fundamental mode to the plane wave obey the Lorentz reciprocity relationship^50^. Substituting Eqs (4)–(6), 5, 6 into Eq. (3), we obtain the equation of HW model to calculate the transmission spectra. When the second term on the right-hand side of Eqs (5) and (6) representing the contribution of the HWs to the EOT is neglected, the equation of No-HW model for the transmission spectra calculation is obtained.

For the calculation using both HW model and RCWA, the temperature dependent permittivity of superconducting NbN film is obtained from calculation based on BCS theory with impurity scattering in the Born limit^51^,^52^. Within the theory, the dielectric function is determined by normal state conductivity (*σ*), scattering rate (*γ*), and *Δ*(0)*/k*_*B*_*T*_*c*_, where *Δ*(0) is the energy gap at 0 K and *k*_*B*_ is the Boltzmann constant. Here, the parameters of *σ*, *γ* and *Δ*(0)*/k*_*B*_*T*_*c*_ are set to be 1.2 × 10^6^ S/m, 40 THz and 2.2 respectively. In our calculations, *n*_*air*_ is approximated to be 1, and *n*_*MgO*_ obtained from the measured THz transmittance of bare MgO substrate is 3.08.

### FDTD simulation

The electric field distribution is simulated using commercial software **FDTD Solutions** based on FDTD method. The permittivity of NbN film is the same as the calculation using HW model. The excitation source in the simulation is a short Gaussian pulse with a frequency range from 0 to 1.5 THz.

## Additional Information

**How to cite this article**: Wu, J. B. *et al*. The dynamic process and microscopic mechanism of extraordinary terahertz transmission through perforated superconducting films. *Sci. Rep*. **5**, 15588; doi: 10.1038/srep15588 (2015).

## Supplementary Material

Supplementary Information
