# Supplementary material for: The dynamic process and microscopic mechanism of extraordinary terahertz transmission through perforated superconducting films
Source: Sci Rep. 2015 Oct 26;5:15588. doi: 10.1038/srep15588 (PMC4620484; doi:10.1038/srep15588)
Supplement: Supplementary Information [file srep15588-s1.doc]

**Supplement Material for “The dynamic process and microscopic mechanism of extraordinary terahertz transmission through perforated superconducting films”**

**J. B. Wu^1,2†^, X. Zhang^3†^, B. B. Jin^1*^, H. T. Liu^3*^, Y. H. Chen^4^, Z. Y. Li^4^, C. H. Zhang^1^, L. Kang^1^, W. W. Xu^1^, J. Chen^1^, H. B. Wang^1,5^, M. Tonouchi^6^, P. H. Wu^1*^**

^1^Research Institute of Superconductor Electronics (RISE), School of Electronic Science and Engineering, Nanjing University, Nanjing 210093, China

^2^School of Electronic and Electrical Engineering, University of Leeds, Leeds LS2 9JT, United Kingdom

^3^Key Lab of Optical Information Science and Technology (MOE), Institute of Modern Optics, Nankai University, Tianjin 300071, China

^4^Laboratory of Optical Physics, Institute of Physics, Chinese Academy of Sciences, Beijing 100190, China

^5^National Institute for Materials Science, Tsukuba 305-0047, Japan

^6^Institute of Laser Engineering, Osaka University, 2-6 Yamadaoka, Suita, Osaka 565-0871, Japan

^*^[bbjin@nju.edu.cn](mailto:bbjin@nju.edu.cn), [liuht@nankai.edu.cn](mailto:liuht@nankai.edu.cn) and [phwu@nju.edu.cn](mailto:phwu@nju.edu.cn)

+ these authors contributed equally to this work


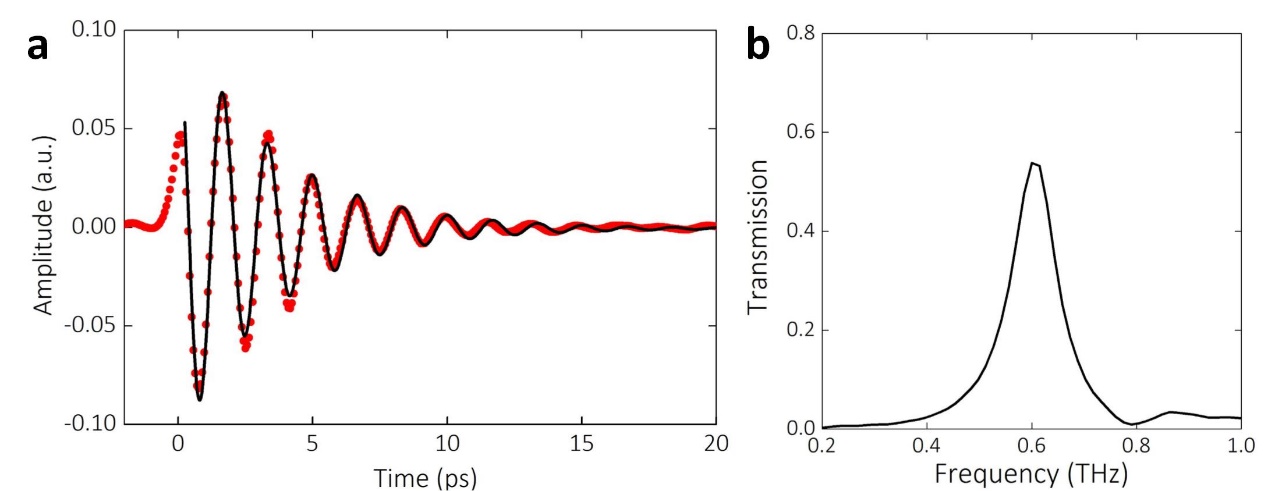


**Supplementary Figure 1.** **The terahertz (THz) tranmission spectra of perorated gold film.** (a) The measured time domain profiles (black dots) of peforated gold film having the same structure as W2.5. The red line is the fitted curve using Eq. (1) of the main article. (b) The correspondingfrequency domain transmission spectra.


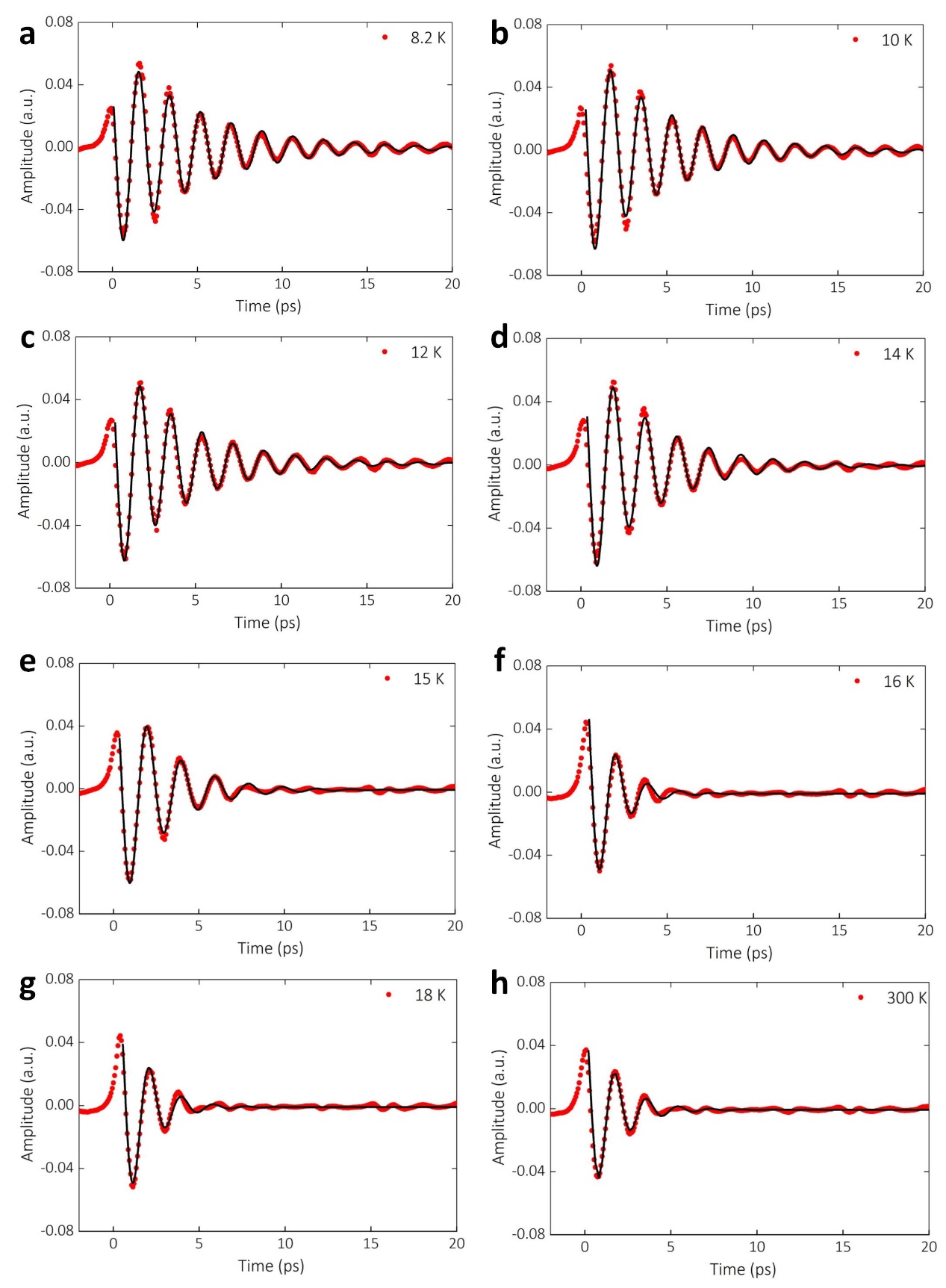


**Supplementary Figure 2. The time domain profies of transmitted pulses through W2.5 at different temperatures.** The measured time domain profiles (red dots) and fitted curves (black lines) of transmitted pulses of W2.5 at temperatures of 8.2 K (a), 10 K (b), 12 K (c), 14 K (d), 15 K (e), 16 K (f), 18 K (g), and 300 K (h).

**Supplementary Note 1. The THz transmssion spectra of perforated gold film.**

We fabricated the subwavelength hole array in gold film and the fabricated sample has the same geometry as W2.5. For the fabrication, first, the complementary structure of subwavelength hole array was patterned onto a 1 mm-thick MgO substrate using photolithography. Then, 10 nm-thick titanium and 200 nm-thick gold films were deposited onto the sample. After lift-off process, the subwavelength hole array was formed in the gold film. The THz transmission spectra of this sample was measured as a comparison. The measured transmitted pulse time domain profile and the fitted curve using Eq. (1) of the main article are shown in Supplementary Fig. 1(a). The fitted curve agrees well with the measured data. The parameters used for fitting are *y*_0_=0.109, *τ_d_* = 3.58 ps, *f* = 0.604 THz, *t*_0_ = -0.439 ps. The lifetime of oscillation is much lower than that of W2.5 at 8.2 K. As shown in Supplementary Fig. 1(b), the transmission peak is only 53.9%, remarkably lower than that of W2.5 in superconducting state.

**Supplementary Note 2. The time domain profies of transmitted pulses through W2.5.**

The measured time domain profies of transmitted pulses through W2.5 at different temperatures (black solid line) are shown in Supplementary Fig. 2. The fitted curves (red dots) using Eq. (1) of the main article are in good accordance with the measured curves. The *f* and *τ_d_* as a function of temperature are obtained from the fitted curves and shown in Fig. 2(b) of the main article.
